# Relation between the Recipe of Yeast Dough Dishes and Their Glycaemic Indices and Loads

**DOI:** 10.3390/foods8090377

**Published:** 2019-09-01

**Authors:** Ewa Raczkowska, Karolina Łoźna, Maciej Bienkiewicz, Karolina Jurczok, Monika Bronkowska

**Affiliations:** Department of Human Nutrition, Faculty of Biotechnology and Food Sciences, Wrocław University of Environmental and Life Sciences, 51-630 Wroclaw, Poland (K.Ł.) (M.B.) (K.J.) (M.B.)

**Keywords:** glycaemic index, glycaemic load, yeast dough

## Abstract

The aim of the study was to evaluate the glycaemic indices (GI) and glycaemic loads (GL) of four food dishes made from yeast dough (steamed dumplings served with yoghurt, apple pancakes sprinkled with sugar powder, rolls with cheese and waffles with sugar powder), based on their traditional and modified recipes. Modification of the yeast dough recipe consisted of replacing wheat flour (type 500) with whole-wheat flour (type 2000). Energy value and the composition of basic nutrients were assessed for every tested dish. The study was conducted on 50 people with an average age of 21.7 ± 1.1 years, and an average body mass index of 21.2 ± 2.0 kg/m^2^. The GI of the analysed food products depended on the total carbohydrate content, dietary fibre content, water content, and energy value. Modification of yeast food products by replacing wheat flour (type 500) with whole-wheat flour (type 2000) contributed to the reduction of their GI and GL values, respectively.

## 1. Introduction

In connection with the growing number of lifestyle diseases, consumers pay increasing attention to food, not only to food that have a better taste but also to food that help maintain good health. Proper nutrition, and especially the enrichment of diet with functional food, might limit the development of many diseases. Cereal products, including bread and dough, should be eaten several times a day due to their high nutritional value and sensory qualities. Confectionery products are food products with high nutritional value (they provide digestible carbohydrates, protein and fat), however, the main factor influencing the willingness to eat them is their taste [1].

A wide range of bakery and confectionery products are food dishes made from yeast dough. Due to their ease of manufacture and taste, dishes from yeast dough are popular in many countries. The basic ingredients used to make the dough are wheat flour, baker’s yeast, water or milk, and salt. These ingredients are sufficient for the production of bread, including wheat bread and rolls. Eggs and fat are added to obtain other products, and sugar and additives (such as lemon peel, raisins and alcohol) are also added to sweet products [2].

Baker’s yeast (Saccharomyces cerevisiae) is the primary leavening agent in the production of yeast dough. Yeast cells consume the fermentable sugars and generate carbon dioxide (CO_2_) and ethanol that are responsible for the dough leavening during fermentation and the oven-rise of the dough. Besides the two primary fermentation metabolites, other secondary metabolites are produced. These metabolites might have an impact on the final product quality. These metabolites determine the taste, smell, colour and even the shelf life of yeast dough dishes [3].

Aroma and flavour are two of the most important parameters for confectionary products. These are mainly affected by ingredients and secondary fermentation products produced by yeast, under baking conditions. These compounds include volatile alcohols, aldehydes and ketones, as well as non-volatile acids, sugars, lipids, esters, phenolic compounds and free fatty acids [4]. The Sahin and Schieberle [5] studies showed that the aroma of yeast dough products consists of at least 29 aroma-active compounds. The most important contributors to the overall aroma of the yeast dough products are 3-methyl-1-butanol (malty), 2-phenylethanol (honey-like), trans-4,5-epoxy-(E)-2-decenal (metallic), 2,3-butanedione (buttery), 3-methylbutanoic acid (sweaty), (E)-2-nonenal (cucumber-like), 1-octen-3-one (mushroom-like) and 3-(methylthio)-propanal (potato-like) [5]. The ability of yeast to produce compounds that shape sensory characteristics, undoubtedly influences the importance and possibility of using yeast dough for the production of bakery products [3,4,5].

The use of Saccharomyces cerevisiae yeast in the production of the dough also influences the pro-health properties of the resulting products [6]. Wheat-based products are a major part of daily-consumed fructans and fructooligosaccharides (FOS), which are the main FODMAP’s (Fermentable Oligosaccharides, Disaccharides, Monosaccharides and Poliols). There is evidence that the intake of FODMAP’s induces pain, nausea, bloating and disturbed bowel habit in people suffering from irritable bowel syndrome (IBS) [7]. It is estimated that 12% of Europeans and 7% to 15% of the world population are affected by this disease. Several studies have showed that the consumption of products with reduced FODMAP’s, alleviates the symptoms of disease in approximately 70% of IBS patients [4,7]. 

Depending on the time of fermentation and the yeast dosage, invertase produced by Saccharomyces cerevisiae cells can hydrolysis between 50% and 80% of the fructans initially present in flour. However, the complete elimination of wheat products from the diet might lead to nutrient deficiencies and, therefore, yeast products might be an alternative [3,7,8].

The challenge for the food industry is to modify the composition of traditional food while maintaining high nutritional value, in response to the needs of some population groups (i.e., children and people at risk of obesity or diabetes) [9,10]. One of the most promising areas for the development of functional foods is the modification of food through the use of various substitutes and health supplements. Diet and nutrition are important factors in maintaining good health and potentially reducing the risk of diseases [11,12,13].

Consumption of bakery products made from whole-grain flour is particularly recommended in the prevention of human diseases, due to the high content of dietary fibre. This type of bread is also considered the most dietetic, is of lower energy value and does not cause overweight [14]. Additionally, sweet breakfast products, such as steamed dumplings, apple pancakes, rolls with cheese and waffles, are popular and likable products eaten as breakfast dishes [15].

The supply of carbohydrates to the organism through food, results in a successive rise of blood glucose concentration that returns to the fasting level after about 2 h since food consumption. Insulin is a hormone responsible for glucose transformation into glycogen and it is stored in the liver. The rate of increase in the blood glucose concentration after ingestion of carbohydrate products is expressed by the glycaemic index (GI). It is defined as the ratio of the area under the curve of glycaemic response measured within 120 min after consumption of a food portion containing 50 g of digestible carbohydrates, to the glycaemic response to the same portion of carbohydrates (50 g) originating from a reference product, usually glucose (GI = 100) [16,17,18].

An important related measure is the glycaemic load (GL), which additionally also accounts for the carbohydrate content of the food. The GI value only indicates how fast the digestible carbohydrates are hydrolysed into glucose and is absorbed, but does not indicate their amount in the product. An indicator that takes into account both, the glycaemic index and the carbohydrate content of commonly consumed portions of products and meals, is the glycaemic load [19]. A growing interest in both GI and GL has been aroused by their association with diseases, such as diabetes, obesity, cardiovascular diseases and neoplasms [20,21,22,23,24,25].

Dishes from yeast dough, like other dishes, are subject to various modifications. Changes are made to traditional formulas to increase the nutritional value or endow additional health-promoting properties. The most common modification is the use of wholemeal flour instead of purified flour or the addition of bran of various cereal grains. The use of cereal grains increases the content of valuable minerals, as well as the content of dietary fibre [26]. Therefore, this study determined the GI and GL of selected dishes made from yeast dough, based on traditional and modified recipes, and how these values are affected by modifying the recipes and composition of the dishes.

## 2. Materials and Methods

### 2.1. Characteristics of the Analysed Dishes

The GI of the dishes were analysed in compliance with the guidelines of the ISO/FDIS 26642:2010 standard “Food products—Determination of the GI and recommendation for food classification” [27], and according to the procedures recommended by the Food and Agriculture Organization of the United Nations (FAO)/World Health Organization (WHO) [28]. The energy value and composition of basic nutrients were determined by the standard analytical methods [29,30]. Energy value was measured by Rozental’s method [29]; water and dry matter contents by the gravimetric (drying) method [30]; total ash content was measured by the gravimetric method after sample incineration in a muffle furnace [30]; protein content was measured through Kjeldahl analysis [30]; and fat content was measured by Soxhlet extraction [30]. The total carbohydrate in the analysed dishes was calculated by difference (100% minus the contents of water, protein, fat and total ash). The dietary fibre content was calculated by summing up its contents in particular products used to prepare the dish, based on food composition and energy value tables [31].

The GI and the GL values were determined for four dishes made from yeast dough (steamed dumplings served with yoghurt, apple pancakes sprinkled with sugar powder, rolls with cheese, and waffles with sugar powder). All dishes were prepared in two versions, traditional—using wheat flour (type 500) and modified—using whole-wheat flour (type 2000). The traditional recipe was taken from “Polish Cuisine” [32].

The serving sizes and basic nutrient compositions determined by the laboratory methods are presented in Table 1. Portions of the analysed dishes contained 50 g of digestible carbohydrates. The content of digestible carbohydrate was computed by deducing the dietary fibre content from the total content of carbohydrate in a product. All products were weighed at the study site, on the same day, and served in identical, white vessels. Preparation of the reference product involved dissolving 50 g of crystalline glucose in 250 mL of warm boiled water, immediately before serving. All dishes were hand-made in a gastronomic unit of the Department of Human Nutrition.

### 2.2. Characteristics of the Surveyed Participants

The survey finally included 50 participants—42 women and 8 men, with an average age of 21.7 ± 1.1 years, and a mean body mass index (BMI) of 21.2 ± 2.0 kg/m^2^. The participants were students of the Wroclaw University of Life Sciences. After the presentation of the study objectives, conditions and course, written consent was obtained from all volunteers who participated in the study. The inclusion criteria were as follows:

- Age—18–40 years;
- No cigarette smoking;
- Good self-perceived health status;
- Not taking drugs that affect carbohydrate metabolism;
- Not using any special diets and preference for diversified food rations;
- Moderate physical activity (no extreme sports activity);
- 10-h night fasting before the study.

The participants were asked to complete a questionnaire concerning their health status, eating habits and taking of drugs. The 24-h diet recall was carried out three times before the study, to allow the assessment of the nutritional patterns, use of any special diets and preference for diversified food rations among the ones surveyed. Individuals pre-classified for the study but whose fasting serum glucose concentration exceeded 99.0 mg/dL and whose health status and drugs taken could affect carbohydrate metabolism disorders were excluded from the study.

Study participants were subjected to the oral glucose tolerance test after the intake of a standard glucose solution (twice, i.e., once at the onset and again at the end of the study), and, respectively, to a single assay of the glycaemic response after the consumption of each analysed dish. Results from these tests were used to compute the GI of all analysed dishes for each surveyed participant. Each analysed dish was consumed by at least 10 participants.

The nutritional status of the participants was evaluated based on anthropometric measurements, including body mass and body height, with the use of an electronic medical scale with a stadiometer (Radwag, Radom, Poland). During the measurements, each person was shoeless, without outwear and was asked to stand up straight. Body mass was measured in fasted persons, with an accuracy of 0.01 kg, while body height was measured with an accuracy of 0.1 cm. Results of body mass and body height measurements were used to compute the BMI, which expresses the ratio of body mass (kg) to the squared body height [m^2^]. Participants were instructed to continue their eating habits and normal physical activity throughout the study. Participants’ characteristics are summarised in Table 2.

The study was approved by the Bioethics Committee of the Medical University in Wroclaw (approval number KB-831/2012).

### 2.3. Course of the Experiment

Measurements of blood glucose concentration obtained, respectively, after drinking the reference solution and consuming the analysed dishes were performed in the morning hours after 10-h night fasting. Before consuming the analysed product, blood was sampled from the participants using an automatic Accu–Chek Softclix lancing device (Roche Diagnostics, Rotkreuz, Switzerland) from a fingertip puncture. Further blood samples were taken at 15, 30, 45, 60, 90 and 120 min, after starting the consumption of the reference solution or a tested dish. Following each blood drop sampling, the blood glucose concentration was measured using an Accu-Chek Active glucometer (Roche Diagnostics). Results were recorded in measuring sheets. The participants had 5–10 min to drink the reference solution and 10–15 min to eat the analysed dishes. Glycaemic curves were plotted twice after drinking the glucose solution and once after consuming the analysed dish. Each participant took part in the determination of the GI of at least one dish, made according to the traditional and modified recipes, respectively. Steamed dumplings and rolls with cheese were consumed by 14 participants, whereas waffles and apple pancakes were consumed by 11 participants.

### 2.4. Calculation of GI and GL

All results of blood glucose concentration measurements after drinking the reference solution and those obtained after consuming the analysed dishes were transferred to an MS Excel 2010 (Microsoft, Redmond, DC, USA) calculation sheet. The area under the glycaemic curves was calculated by dividing the curves into triangles and trapezoids. Negative results of area size were not considered in the calculations. GI and GL values were computed by the following equations:

(1) $$\mathrm{GI}\;\mathrm{of}\;\mathrm{analysed}\;\mathrm{dish}=\frac{\mathrm{Area}\;\mathrm{under}\;\mathrm{glycaemic}\;\mathrm{curve}\;\mathrm{for}\;\mathrm{dish}}{\mathrm{Area}\;\mathrm{under}\;\mathrm{glycaemic}\;\mathrm{curve}\;\mathrm{for}\;\mathrm{glucose}}\;\times 100,$$

(2) $$\mathrm{GL}\;\mathrm{of}\;\mathrm{analysed}\;\mathrm{dish}=\frac{C\;\times \;\mathrm{GI}}{100},\;$$

where *C* is the content of digestible carbohydrates in the analysed food portion.

When the individual GI values for any subject fell outside the range of values calculated as mean ± (2 × standard deviation), this result was considered as an outlier and was excluded from the mean GI calculation.

### 2.5. Statistical Analysis

The results were statistically evaluated using Statistica 10 PL (StatSoft, Tulsa, OK, USA). The normality of the distribution of variables was checked by the Shapiro–Wilk test. To show statistically significant differences in the GI and GL of dishes between the traditional and modified versions, the Student’s *t*-test for independent groups was performed. Values of the coefficient of variability (CV) of glycaemia after a 2-fold intake of the standard glucose solution did not exceed 15% (the required value is CV < 30%). For assessing the relationship between the energy value of each dish, its ingredient composition (protein, fat, carbohydrate, water, total ash) and its GI, Pearson’s correlation analysis was performed. Statistical significance was set at *p* < 0.05.

## 3. Results

### 3.1. Chemical Composition of Meals

Table 1 presents the energy value and nutrient composition per 100 g of the analysed dishes, prepared according to the traditional and modified recipes, as well as the portion sizes of the dishes served. Dishes made according to the modified recipe were characterised by a lower energy value, lower content of total carbohydrates and higher contents of protein, total ash and water, compared with their traditional equivalents. As expected, the modification of recipes increased the dietary fibre content, around two-fold in each case. Waffles presented the highest energy value of 318.8 and 308.7 kcal/100 g in the traditional and modified version, respectively. Total carbohydrates ranged from 34.9 g/100 g for modified pancakes with apples to 43.8 g/100 g for traditional rolls with cheese. Table 1 also shows the serving sizes containing 50 g of digestible carbohydrates. Portions of modified dishes were higher due to the increased content of dietary fibre.

### 3.2. GI, GL and Glycaemic Response Curves of Test Meals

**Figure 1:**
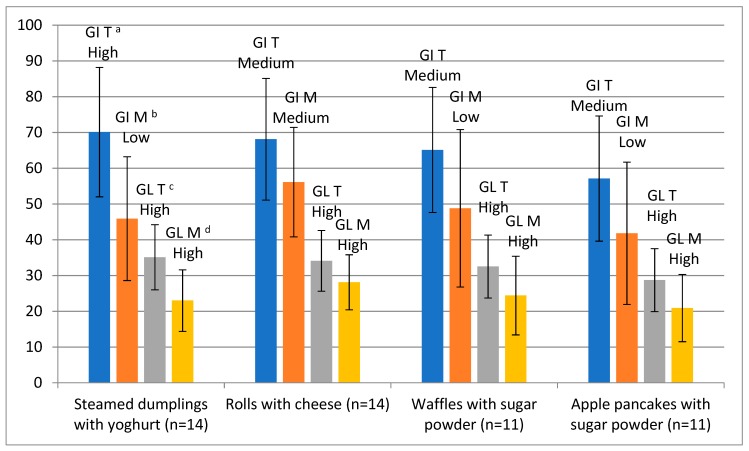
Glycaemic index (GI) and glycaemic load (GL) of the examined dishes. GI T/GL T—Glycaemic Index and Glycaemic Load of the traditional version; GI M/GL M—Glycaemic Index and Glycaemic Load of the modified version; ^a,b,c,d^ statistically significant differences determined by the Student’s *t*-test at *p* < 0.05 (^a,b^ GI, ^c,d^ GL). GI values were classified as high (≥70), medium (56–69) and low (≤55) [27]; GL values were classified as high (≥20), medium (11–19) and low (≤10) [18].

Mean values of GI and GL of the analysed dishes are summarised in Figure 1. Among the dished prepared from the traditional recipes, the highest GI was determined for the steamed dumplings—70.1, whereas, the lowest GI was obtained for pancakes with apples—57.4. Regarding the modified dishes, the highest GI was assayed for rolls with cheese—56.1, and, again, pancakes with apples obtained the lowest GI—41.8. The GL of the traditional dishes ranged from 28.7 (pancakes with apples) to 35.1 (steamed dumplings), and 20.9 (pancakes with apples) to 28.1 (rolls with cheese) for the modified dishes. In all cases, the modification of the recipe caused a decrease in the GI and GL, but statistically significant differences were observed only after consumption of steamed dumplings served with yoghurt. All traditional dishes (except steamed dumplings with yoghurt) had a medium GI, while all modified dishes had a low GI, except for rolls with cheese, which had a medium GI. The GL of all dishes was deemed high. The GL of the different tested foods was calculated, considering the portions reported in Table 1.

**Figure 2:**
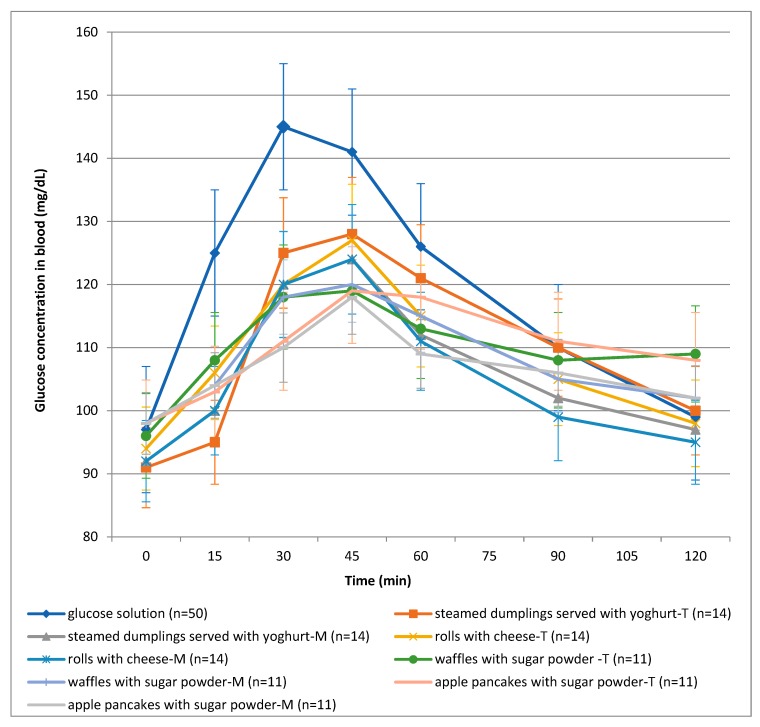
Curves of mean glycaemic response after consumption of a standard glucose solution and meals. (T = traditional version; M = modified version).

Figure 2 shows the curves of mean glycaemic response after ingestion of the reference glucose solution and the tested meals, respectively. The maximal blood glucose level appeared 30 min after drinking the reference solution (145 mg/dL) as compared to 45 min after consuming the analysed dishes. Maximal mean blood glucose concentrations ranged from 118 to 128 mg/dL after the consumption of the traditional dishes, and from 118 to 124 mg/dL after the consumption of the modified dishes. The mildest course of the curve of the average glycaemic response was plotted after the consumption of pancakes with apples. A comparison of particular glycaemic responses determined 15, 30, 45, 60, 90 and 120 min after the consumption of dishes prepared based on traditional and modified recipes showed no statistically significant differences.

Correlations between the energy value of dishes, contents of their components (protein, fat, carbohydrate, water, total ash) and their GI were determined on the basis of Pearson’s correlation analysis (Table 3). The GI showed a significant positive correlation with the carbohydrate content (*r* = 0.410) and the energy value of the dishes (*r* = 0.241), and a significantly negative correlation with the contents of dietary fibre (*r* = −0.440) and water (*r* = −0.271), respectively.

The increased contents of dietary fibre and water probably contributed most to the observed reduction in the GI of the modified dishes as compared to their traditional versions. The composition of a food product is related to its energy value. Hence, lower energy value is accompanied by a lower GI. The carbohydrate content is also significant, since the higher its value, the higher the GI of a dish.

## 4. Discussion

GI and GL data obtained from numerous investigations of food products have been collated in the form of international tables. This table contains products and dishes typical of the countries participating in these investigations [33,34]. The benefits of using the values of GI and GL in the planning of diets in many diseases should be the reason of constant expansion of this base to include foods popular in the nutrition of various societies.

There are only few studies on glycaemic response after digestion pastry products but most concern other types of dough, such as shortbread dough or sponge cake [35,36,37]. There are no studies on sweet products prepared from yeast dough and, therefore, this study is desirable. 

There are several studies in which the influence of wheat and wholegrain bread consumption on the glycaemic response was studied. In these studies, just like in the present study, it was shown that after the consumption of wholegrain bread, the glycaemic response was lower in comparison to the glycaemic response after the consumption of bread prepared from purified flour (Figure 2) [38,39].

Additionally, there are several papers in which other types of dough were modified by replacing wheat flour with whole grain flour. As in the present study, such a modification was applied by Bae et al. [40], who investigated the effect of whole-grain flour content in a recipe on the quality and GI of the prepared dough. The GI was estimated based on in vitro starch digestion rate. Doughs made of whole-grain flour (whole-wheat and buckwheat) were characterised by a significantly lower GI than those prepared from wheat flour, and additionally, doughs with 50% contribution of whole-grain flour preserved the desired volume and texture [40]. Similar investigations were conducted by Ferrer–Mairal et al. [41] on the GI of two bakery products (bread and muffins) determined in vivo with the participation of 18 volunteers, and by in vitro assay of the starch digestion rate. The results proved that partial substitution of wheat flour with a mixture of resistant starch, dextrin and lentil flour significantly reduced the GI of the products. This trend was similar in both methods applied (Figure 1) [41].

Based on the analysis of the results of our own research, it was shown that in the case of the tested foods, the glucose concentration decreased during the period of 45–120 min into the test. An interesting phenomenon was observed after the consumption of waffles with sugar-powder-T. After consuming this dish, lower glucose levels were observed after 90 min as compared to after 120 min into the test (Figure 2). It can be assumed that a renewed increase in blood glucose levels is a consequence of the addition of sugar powder and content of nutrients such as fat and dietary fibre. After the consumption of waffles with sugar powder-T, the observed (from 0 to 45 minute of the test) increase in blood glucose levels could be caused by the decomposition of sucrose to fructose and glucose. Insulin secretion reduced blood glucose levels within 90 min of the test. Waffles with sugar-powder-T is a product that contained more than 11 g of fat, which could delay the action of digestive enzymes. Therefore, we assumed that an increase in blood glucose after 90 min is the body’s response to the carbohydrates released from the product. This phenomenon was not observed in the modified waffles (waffles with sugar-powder-M). This product contained twice as much fibre as traditional waffles, and it might have increased the time needed to digest the carbohydrates present in the dough. The influence of dietary fibre and fat on the reduction of glucose levels in blood was also investigated by Borczak and Taye [39,42].

Litwinek et al. [43] attempted to modify the GI and GL of biscuits by replacing wheat flour type 650 with oatmeal flour. The use of oatmeal flour significantly increased the crude fat, ash and dietary fibre in the product, and all products had a low GI (46–50) and GL (<10) [43].

In a comparison to the glycaemic responses recorded after consumption of bread and biscuits made form a traditional recipe with those modified by introducing a dietary fibre mixture (70% inulin, 20% guar gum, 5% glucomannan and 5% wheat fibre) to the applied flour, Marangoni and Poli [44] and Taye [42] demonstrated that the fibre-enriched products had a lower GI [42,44]. Our own research also showed that the replacement of wheat flour with wholegrain wheat flour resulted in an increase in the content of dietary fibre and total ash in the studied dishes (Table 1). It had an impact on the reduction of the GI value of modified dishes. The GI of the dishes also decreased as a result of increasing the protein and fat in the dish. Elsewhere, Henry et al. [45] proved that the addition of sauces/fillings (Cheddar cheese, chilli con carne, baked beans and tuna) reduced the glycaemic response to potatoes, pasta and toast. The incorporation of Cheddar cheese had the greatest impact on the GI, as this product is rich in fat and protein. The presence of fat in the product delays stomach emptying, which, in turn, slows the absorption of carbohydrates [45]. Similar to this study, Kouamé et al. [46] studied the GI and GL of some street foods prepared from plantain and showed a significant inverse relationship between GI and total carbohydrate content, while there was no correlation between GI and protein content in the studied foods [46]. Results of own studies and the above-cited discussions confirmed that food products made with the use of whole-grain raw materials or with the addition of mixtures with a high content of dietary fibre had a lower GI than dishes made of flour from purified grains. Products with a higher content of dietary fibre are less susceptible to the action of digestive enzymes, which results in their lower GI compared with highly-purified products [47].

## 5. Conclusions

The results of these studies showed that the replacement of wheat flour with wholegrain wheat flour decreased the total carbohydrate content and reduced the energy value of the tested products. Modification of the recipes also caused an increase in the protein and dietary fibre content. Correlation analysis showed that the GI value significantly increases with the increase in carbohydrate content and the energy value of dishes. The components significantly influencing the reduction of GI value are dietary fibre and water. The results of our research provide the first GI values of several dishes made from yeast dough.

Modification of the yeast food products by replacing wheat flour (type 500) with whole-wheat flour (type 2000) contributed to the reduction in the GI value. Although the modification of the recipes also reduced the GL value, the value of this indicator was considered high for all dishes. The results obtained in the framework of this work could help to assess the impact of modified dishes made of yeast dough, commonly consumed in different countries, on the glycaemic response. Additionally, obtained results might significantly facilitate the conscious planning of nutrition, depending on the state of health.

## Figures and Tables

**Table 1:** The energy value and nutrient composition of the investigated meal (per 100 g) and that provided in portion size of 50 g digestible carbohydrates.

Recipe	Steamed Dumplings with Yoghurt		Rolls with Cheese		Waffles with Sugar Powder		Apple Pancakes with Sugar Powder	
	T	M	T	M	T	M	T	M
	X ± SD	X ± SD	X ± SD	X ± SD	X ± SD	X ± SD	X ± SD	X ± SD
Energy value (kcal/100 g)	260.3 ± 8.3	244.7 ± 9.3	294.6 ± 15.9	260.2 ± 12.3	318.8 ± 7.9	308.7 ± 8.8	229.7 ± 11.5	212.6 ± 7.8
Protein (g/100 g)	7.5 ± 0.3	8.4 ± 0.6	10.7 ± 0.4	12.6 ± 0.5	9.9 ± 0.5	10.4 ± 0.7	6.5 ± 0.3	7.3 ± 0.3
Total carbohydrates (g/100 g)	38.9 ± 3.6	35.5 ± 3.8	43.8 ± 4.8	40.6 ± 5.1	41.5 ± 3.2	36.9 ± 3.5	39.5 ± 3.7	34.9 ± 3.2
Fat (g/100 g)	6.2 ± 0.4	5.9 ± 0.4	5.9 ± 0.6	6.6 ± 0.4	11.9 ± 0.7	11.7 ± 0.6	3.6 ± 0.4	3.8 ± 0.3
Fibre (g/100 g)	1.1	2.7	1.4	3.2	1.2	2.8	1.4	2.6
Total ash (g/100 g)	0.57 ± 0.03	0.93 ± 0.05	0.94 ± 0.05	1.48 ± 0.08	0.81 ± 0.04	1.18 ± 0.05	0.59 ± 0.03	0.78 ± 0.04
Water (g/100 g)	46.8 ± 2.3	49.3 ± 1.4	38.6 ± 1.3	38.7 ± 1.9	35.6 ± 1.7	39.8 ± 1.8	49.8 ± 2.2	53.2 ± 2.0
Test portion size (g) ^1^	133.0	153.0	119.0	135.0	125.0	148.0	132.0	156.0

^1^ Test portion providing 50 g of carbohydrates. T = traditional version; M = modified version; X ± SD = mean ± standard deviation.

**Table 2:** Baseline clinical and anthropometric characteristics (mean ± standard error of the mean (SEM)) of participants (*n* = 50) involved in the study.

Parameter	Sample		
	Mean	SEM	Range
Age (years)	21.7	1.1	21.0–27.0
Gender (female/male)	42/8		
Body weight (kg)	60.3	8.5	49.2–87.0
Height (m)	1.68	0.1	1.6–1.9
BMI (kg/m^2^)	21.2	2.0	19.1–24.9
Fasting glucose (mg/dL)	93.0	0.1	81.0–98.0

BMI = body mass index.

**Table 3:** Pearson’s correlation coefficients between the glycaemic index (GI) and energy value, nutrient composition of the examined dishes.

Correlation	Pearson’s Correlation Coefficient (*r*)
GI and energy value	0.241 *
GI and protein content	0.068
GI and fat content	0.057
GI and total carbohydrate content	0.410 *
GI and the ash content	−0.140
GI and fibre content	−0.440 *
GI and water content	−0.271 *

* *p* < 0.05.
